# Metformin repositioning as antitumoral agent: selective antiproliferative effects in human glioblastoma stem cells, via inhibition of CLIC1-mediated ion current

**DOI:** 10.18632/oncotarget.2617

**Published:** 2014-10-21

**Authors:** Marta Gritti, Roberto Würth, Marina Angelini, Federica Barbieri, Marta Peretti, Erika Pizzi, Alessandra Pattarozzi, Elisa Carra, Rodolfo Sirito, Antonio Daga, Paul M.G. Curmi, Michele Mazzanti, Tullio Florio

**Affiliations:** ^1^ Dipartimento di Bioscienze, University of Milano, Italy; ^2^ Sezione di Farmacologia, Dipartimento di Medicina Interna & Centro di Eccellenza per la Ricerca Biomedica (CEBR), University of Genova, Italy; ^3^ Laboratorio di Trasferimento Genico, IRCCS-AOU San Martino-IST, Genova, Italy; ^4^ Dipartimento di Ostetricia e Ginecologia, Ospedale Evangelico Internazionale, Genova, Italy; ^5^ School of Physics, University of New South Wales, Sydney, Australia

**Keywords:** Metformin, Cancer Stem Cells, Human Glioblastoma, CLIC1, Antitumoral Activity

## Abstract

Epidemiological and preclinical studies propose that metformin, a first-line drug for type-2 diabetes, exerts direct antitumor activity. Although several clinical trials are ongoing, the molecular mechanisms of this effect are unknown.

Here we show that chloride intracellular channel-1 (CLIC1) is a direct target of metformin in human glioblastoma cells. Metformin exposure induces antiproliferative effects in cancer stem cell-enriched cultures, isolated from three individual WHO grade IV human glioblastomas. These effects phenocopy metformin-mediated inhibition of a chloride current specifically dependent on CLIC1 functional activity. CLIC1 ion channel is preferentially active during the G1-S transition via transient membrane insertion. Metformin inhibition of CLIC1 activity induces G1 arrest of glioblastoma stem cells. This effect was time-dependent, and prolonged treatments caused antiproliferative effects also for low, clinically significant, metformin concentrations. Furthermore, substitution of Arg29 in the putative CLIC1 pore region impairs metformin modulation of channel activity.

The lack of drugs affecting cancer stem cell viability is the main cause of therapy failure and tumor relapse. We identified CLIC1 not only as a modulator of cell cycle progression in human glioblastoma stem cells but also as the main target of metformin's antiproliferative activity, paving the way for novel and needed pharmacological approaches to glioblastoma treatment.

## INTRODUCTION

Epidemiological studies reported that metformin, a first-line treatment for type-2 diabetes [1], is associated with reduced incidence and favorable prognosis in several cancers [2-4]. Moreover, metformin directly inhibits cancer cell proliferation, mainly acting on cancer stem cells (CSCs) [5-10]. On these bases, several clinical trials are underway [4, 11].

Although metformin was clinically approved several decades ago, its mechanism of action has not been completely elucidated. Metformin metabolic effects mainly rely on mitochondrial activity: it decreases ATP production and activates AMP-activated protein kinase (AMPK), thus regulating gluconeogenesis and fatty acid synthesis [12]. Since AMPK controls mammalian target of rapamycin (mTOR) activity, metformin's regulation of AMPK could also account for its antiproliferative effects [13, 14].

However, several lines of evidence suggest that this is not the case. If metformin merely acted as mTOR inhibitor, it should induce the same tumor resistance mechanisms as “classical” mTOR blockers (*i.e*. rapamycin), such as the relief of the mTOR/S6K1 negative feedback loop on IGFR-1/IRS-1, the activation of receptor tyrosine kinase-mediated intracellular pathways [15] and the loss of antiproliferative activity [16]. In contrast, epidemiological and preclinical studies suggest that metformin has long-term antiproliferative effects. Further, in CSCs isolated from breast and lung carcinomas, or glioblastoma (GBM) [8, 9, 17-21], metformin-dependent cell proliferation arrest does not involve AMPK, but rather the down-regulation of IGF-1 signaling or inhibition of Akt [17, 22, 23]. Conversely, the synthetic AMPK agonist A-769662 provided a proliferative advantage to the cells [21]. On the other hand, metformin was reported to affect several other intracellular pathways in tumor cells, including HER1/HER2, Src, S6K1, c-MYC, and STAT3 among others [16, 24-27], being also able to overcome dietary restriction-resistance in cancer cells [28].

The heterogeneity of the reported mechanisms of action could imply that metformin is a promiscuous drug, rather than acting on precise pathways. Conversely, the broad range of tumors affected by metformin might suggest that, instead of modulating different pathways in each cancer histotype, an upstream target could represent its specific mechanism of action.

In this context, our study aims to identify the molecular mechanism by which metformin specifically elicits antitumoral effects without interfering with normal cell viability.

Chloride intracellular channel 1 (CLIC1) [29-31] is involved in development of the most aggressive human tumors, including GBMs [32-34]. In resting cells, CLIC1 is mainly localized in the cytosol, but it is progressively oxidized during cell cycle progression, and transiently recruited to the plasma-membrane, where it functions as a chloride selective ion channel [35, 36]. *In vivo* and *in vitro* proliferation of GBM CSCs depends on CLIC1 activity and its inhibition reduces tumor development in animal models [32], thus, CLIC1 could be a target for antiproliferative molecules.

Importantly, *in vivo* studies already demonstrated that CLIC1 is required for GBM tumorigenesis [32], and that metformin treatment of mice orthotopically xenografted with human GBM CSCs, reduced tumor growth [18], confirming the more copious *in vitro* results.

On these premises, the goal of this study was to determine whether CLIC1 is involved in metformin inhibition of GBM cell proliferation.

## RESULTS

### Correlation between CLIC1 inhibition and antiproliferative effect of metformin in glioblastoma cells

**Fig. 1:**
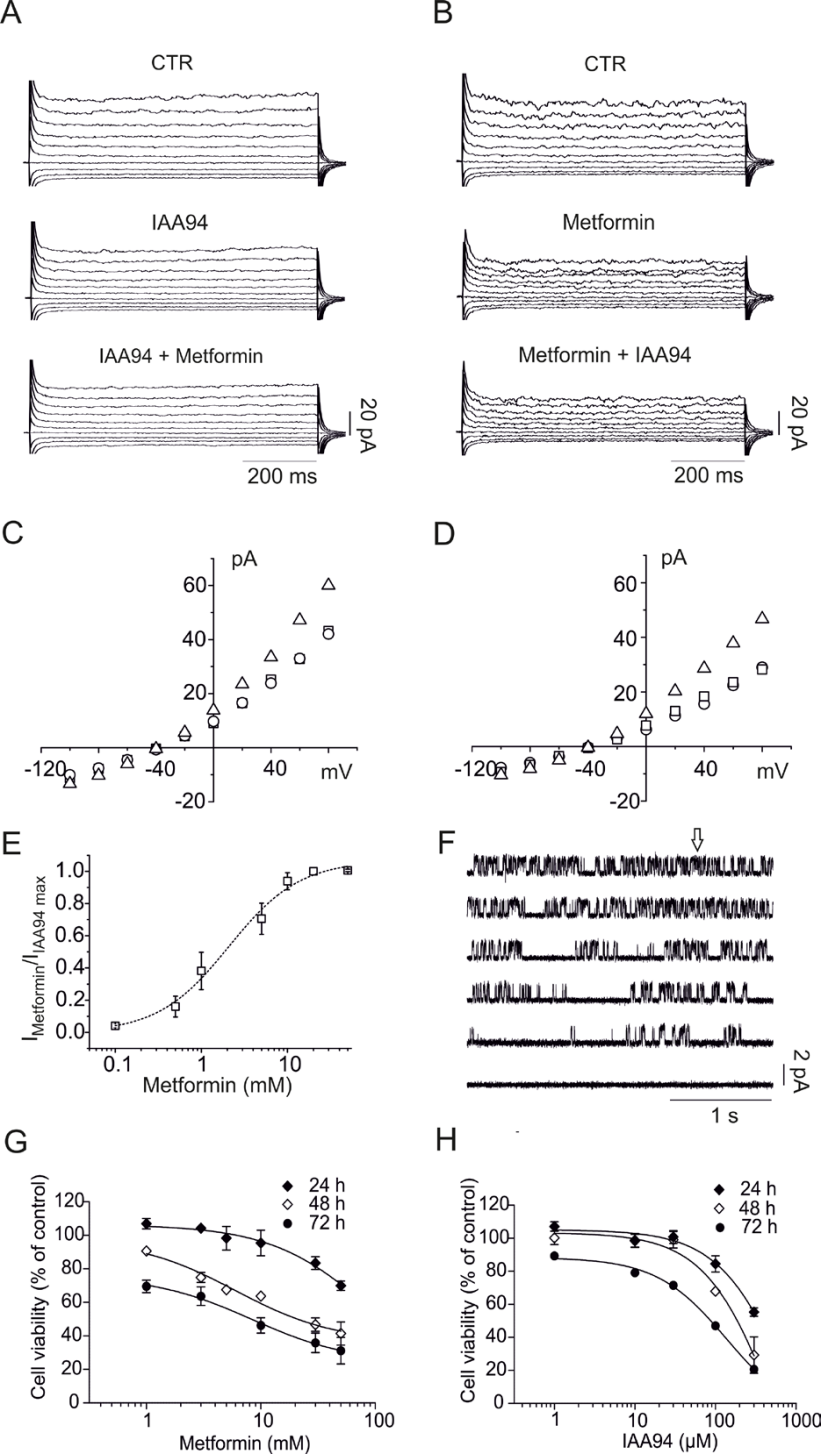
Metformin decreases U87 cell viability via CLIC1 inhibition A) Representative family of membrane currents elicited by 800 ms voltage steps from −100 to +80 mV, starting from a holding potential of −40 mV, is shown in the control condition (top), after perfusion of IAA94 (100μM, middle) and after addition of metformin (10mM, bottom) and *vice versa* (B). C) Current/voltage relationships from the data in A, in which IAA94 is perfused alone or followed by metformin, and *vice versa* (D). Total current (triangles) is plotted together with the current amplitude after IAA94 (circles) perfusion or IAA94 and metformin (squares). E) The ratio between metformin and IAA94 sensitive currents (+80 mV, 750 ms test potential) was used to calculate a metformin EC_50_ of 2.1 ± 0.4 mM (mean ± s.e.m.) from a dose/response plot. (n=4 independent experiments for each concentration). F) Ten mM metformin (arrow) caused inhibition of CLIC1 single-channel opening in outside-out experiments, recorded at +60 mV holding potential (single channel in control conditions P_open_= 0.36±0.012, 0.28±0.08 and 0.09±0.07 between 5 and 10 and 10 and 15 seconds after metformin addition, respectively; n=3 total of 4 minutes continuous recording). G-H) Time- and dose-dependent effects of metformin and IAA94 on U87 cell survival evaluated by MTT assay. Experiments were run in quadruplicate and the percentage of inhibition calculated against vehicle control. Data are expressed as mean ± s.e.m of n=3 independent experiments. Statistical significance is reported in Table 1.

Metformin effects were initially tested in U87 human GBM cell line. We measured the effects of IAA94, a well-characterized CLIC1 inhibitor [31], prior or subsequently to the addition of metformin, in perforated patch clamp whole-cell configuration experiments. In both cases, the first compound decreased the whole cell current that was not further reduced by the second one (Fig. 1A and B). Current/voltage (I/Vs) relationships (Fig. 1C and D) show that the current amplitudes, at different membrane potentials, are superimposed, suggesting that the two drugs converge on the same molecular target (Fig. S1). Metformin EC_50_, as CLIC1 inhibitor, was 2.1mM (Fig. 1E), while IAA94 showed EC_50_ (32μM, Fig. S1D) similar to previous reports [31].

**Table 1:** Statistical significance of time- and dose-dependent effects of metformin and IAA94 on U87 cell survival

Time	Metformin (mM)					IAA94 (μM)				
	1	3	10	30	50	1	10	30	100	300
24h	n.s.	n.s.	n.s.	<0.0001	<0.0001	n.s.	n.s.	n.s.	<0.0001	<0.0001
48h	<0.05	<0.0001	<0.0001	<0.0001	<0.0001	n.s.	n.s.	n.s.	<0.0001	<0.0001
72h	<0.0005	<0.0001	<0.0001	<0.0001	<0.0001	n.s.	n.s.	n.s.	<0.0001	<0.0001

Data are obtained comparing treated cell viability with vehicle-treated control cells, in MTT assay.

One-way ANOVA, followed by Dunnett's post hoc test.

n.s.: not significant.

Outside-out single-channel recordings confirmed CLIC1 as metformin target on U87 membranes, where CLIC1 retains single-channel properties, previously described for outside-out experiments (Fig. S2A and B) [29]. Metformin perfusion (Fig. 1F, arrow) efficiently inhibits single channel activity, showing a current inhibition that lasted for several minutes after wash-out, being practically irreversible (Fig. S2C), and highly specific for CLIC1, since 4,4′-diisothiocyanatostilbene-2,2′-disulphonic acid (*DIDS)-*sensitive chloride channels were unaffected (Fig. S2D).

Inhibition of CLIC1 current by metformin or IAA94 was associated with time- and dose-dependent decrease of U87 cell viability, showing maximal efficacy after 72 hours of treatment (MTT assay; Fig. 1G and H, Table 1). EC_50_ values (1.7mM for metformin and 40.5μM for IAA94) are comparable to those obtained in electrophysiology experiments (see above), suggesting that the inhibition of CLIC1 activity and the reduction of cell viability are related events. Metformin efficacy as antiproliferative agent was also time-dependent since longer exposure to the drug required lower metformin concentrations to elicit antitumoral effects (EC_50_: 23, 6.6 and 1.7mM after 24, 48 and 72 hours).

### CLIC1 involvement in metformin antiproliferative effects in human glioblastoma cancer stem cells

**Fig. 2:**
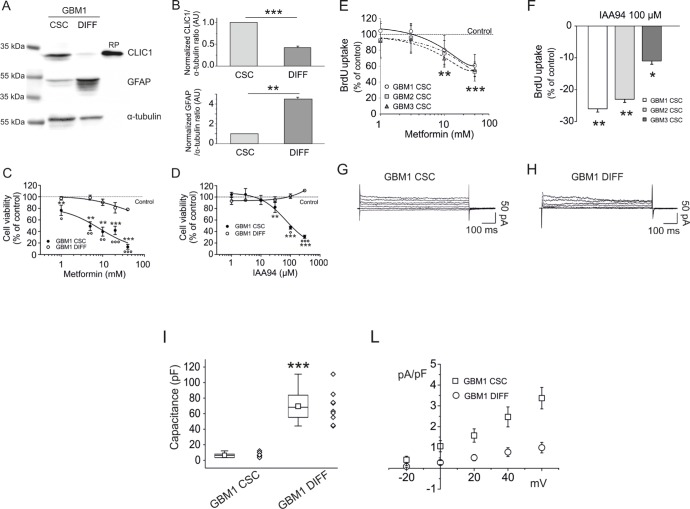
Metformin reduces human GBM CSC viability *via* inhibition of CLIC1 current A) Representative expression of CLIC1 and GFAP in GBM1 CSC-enriched and differentiated cell cultures, obtained shifting CSCs from growth factor-supplemented to growth factor-free and FBS-containing medium. CLIC1 expression is reduced in differentiated cells, which conversely show increased GFAP expression. Similar results are obtained in GBM 2 and 3 (Fig. S4). RP = recombinant CLIC1 protein (positive control). B) Quantification of the data in A, by densitometry, averaging replica Western blots (n=3). An inverse modulation of CLIC1 and GFAP expression occurs after GBM1 cell differentiation. Band intensities were normalized to the corresponding α-tubulin signal and expressed as arbitrary units (A.U.) referred to CSC protein levels (assumed as 1). **p<0.01; ***p<0.001 (t-test). C) Metformin dose-response curves (1-40mM) on GBM1 CSC (filled circles) and differentiated (empty circles) cell viability, measured by MTT assay. Experiments, were performed in quadruplicate (n=3); percentage of inhibition was calculated *vs.* vehicle-treated controls. A significant cell viability reduction was observed in CSCs (**p<0.01 and ***p<0.001 *vs*. controls, one-way ANOVA, followed by Dunnett's test). In differentiated cells a modest effect was observed (**p<0.01 *vs.* controls). At all concentrations, metformin effects on CSCs were statistically different from those induced in differentiated cells (°p<0.05, °°p<0.01, °°°p<0.001 *vs.* differentiated cells; one-way ANOVA, Tukey's test). Dotted line represents vehicle-treated control values. D) Dose-response effects of IAA94 (1-300μM) on viability of GBM1 CSC (filled circles) and differentiated (empty circles) cells, measured by MTT assays. Experiments were run in quadruplicate, and the percentage of inhibition was calculated against vehicle controls (n=3). A significant inhibition of cell viability was observed. (**p<0.01 and ***p<0.001 *vs*. controls, one-way ANOVA, followed by Dunnett's test), while differentiated cells were not affected. IAA94 effects on CSCs were also statistically different from those observed in differentiated cells (°p<0.05, °°°p<0.001 *vs.* differentiated cells; one-way ANOVA, followed by Tukey's test). Dotted line represents the respective vehicle-treated control values. E) Metformin (1-30mM) inhibition of DNA synthesis in GBM 1-3 CSCs, measured by BrdU incorporation assay. A significant reduction of DNA synthesis was observed after 24 hours of treatment of randomly cycling CSCs (**p<0.01 and ***p<0.001, *vs*. respective controls; one-way ANOVA, followed by Dunnett's test). Dotted line represents vehicle-treated control values. F) IAA94 (100 μM) inhibits DNA synthesis in GBM 1-3 CSCs, measured by BrdU incorporation assay. A significant reduction in DNA synthesis was observed after 24 hours of treatment of randomly cycling CSCs (*p<0.05 and **p<0.01 *vs*. respective controls, one-way ANOVA, followed by Dunnett's test). G-H) Comparison of metformin-sensitive membrane current recorded in perforated patch experiments, elicited by 20 mV and 800 ms voltage steps from −40 mV holding potential to +60 mV in CSC (G) and differentiated (H) GBM1 cells. I-L) Cell capacitance measurements (n=10, GBM1 CSCs mean=6.52+/−0.92; GBM1 differentiated cells mean=69.56+/−6.46, ***p<0.001, t-test) (I), were instrumental to obtain membrane current densities (n=5, mean ± s.e.m. from 5 independent experiments (L). While differentiated cells show a 10-fold larger membrane extension, they have one-third of the metformin-sensitive current density compared to CSCs (p<0.001, t-test).

GBM CSC-enriched cultures better reproduce *in vitro,* the biological and pharmacological *in vivo* behavior of tumor cells than established cell lines [37, 38]. We isolated CSCs from three human GBMs, to test the role of CLIC1 in the antiproliferative effects of metformin. These cells were either grown in stem cell-permissive medium [39] retaining CSC-like features (clonogenicity, stem cell marker expression, and *in vivo* tumorigenicity), or shifted for 14-days in FBS-containing medium to induce differentiation (Fig. S3A). Differentiation was demonstrated by increased expression of astrocytic (GFAP: Fig. 2A and B, and Fig. S4C and E) and neuronal (βIII-tubulin: Fig. S3B and C) markers, and the parallel down-regulation of stem cell makers (Nestin, Olig2, and Sox2: Fig. S3B and C).

CLIC1 was highly expressed in CSC cultures, but its protein levels were highly down-regulated after differentiation (Fig. 2A and B, and Fig. S4A, B, C and D). Metformin dose-dependently reduced CSC viability (EC_50_: 3.9, 11.3, and 8.0mM for GBM1-3, respectively, after 48 hours of treatment), but failed to induce cytotoxicity in differentiated cells (Fig. 2C, and Fig. S4F) that resulted statistically significant only at highest concentration (40mM), reaching a maximal inhibition <30%, while the same concentrations almost completely suppress CSC viability (−76-86% of cell viability) (Fig. 2C and Fig. S4F). Similar results were obtained blocking CLIC1 activity using IAA94 (Fig. 2D, and Fig. S4G). The analysis of DNA synthesis, by BrdU incorporation assay, confirmed the antiproliferative activity on CSC by both metformin and IAA94 (Fig. 2E and F).

The correlation of metformin's inhibition of CSC proliferation and CLIC1 functioning, was corroborated by electrophysiological measurements of metformin-sensitive CLIC1 current densities that were negligible in differentiated cells as compared to CSCs (Fig. 2G, H, I and L).

**Fig. 3:**
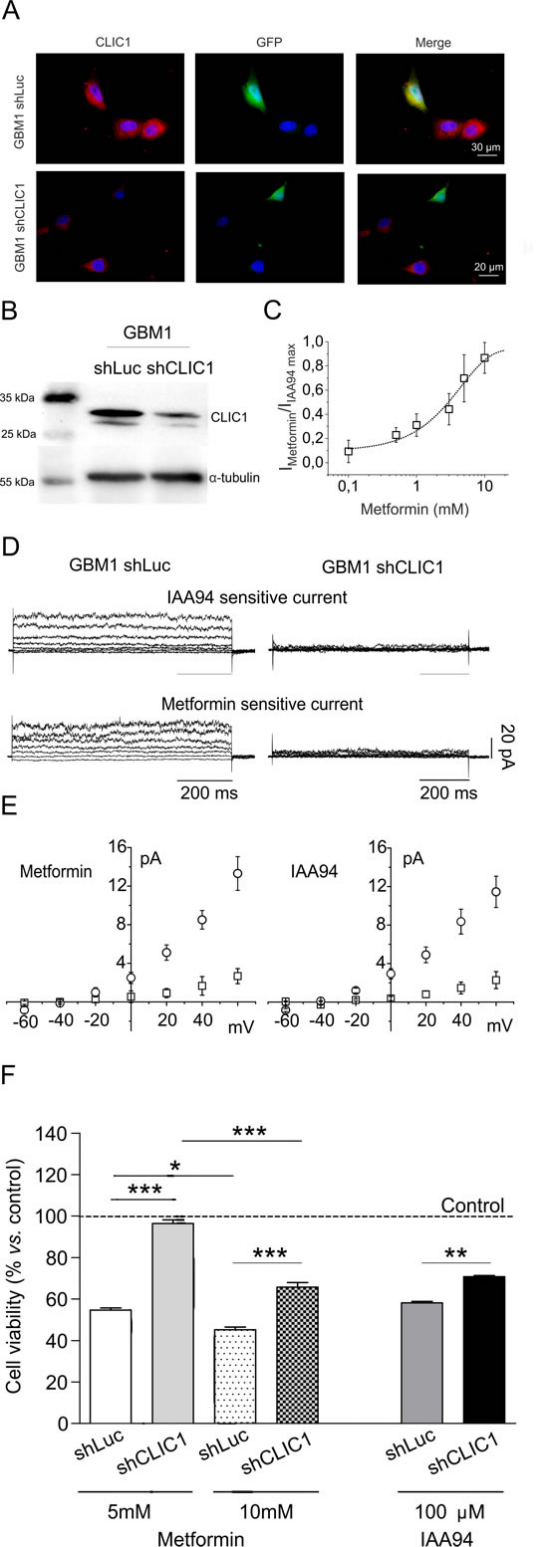
CLIC1 silencing increases human GBM CSC survival in the presence of metformin A and B) GBM1 CSCs were transiently infected with a plasmid carrying validated shRNA for both Luciferase (shLuc, as a silencing control) and CLIC1 (shCLIC1) and tested for CLIC1 expression. In shCLIC1 cells, protein levels were highly reduced as assessed by immunocytofluorescence (A, shRNA= green, CLIC1= red) and Western blot analysis (B). Reported blot is representative of 2 independent experiments. C) Metformin inhibition of CLIC1 current in GBM1 CSCs. Dose-response of metformin sensitive current experiments expressed as a percentage of maximal inhibition operated by 100 μM IAA94 (n=3 for 0.1-5-20 mM, n=6 for 1 mM, n=4 for 10 mM), an EC_50_ of 2.3 mM. D) Perforated patch whole-cell experiments using a voltage protocol of 800 ms, 20 mV steps from −60 to +60 mV, starting from −40 mV holding potential. Left panels depict IAA94- and metformin-sensitive currents in shLuc cells. In shCLIC1-transfected cells (right panels), the current that results sensitive to either metformin or IAA94 treatment is null. E) I/V relationships of metformin (left panel) and IAA94 (right panel) sensitive current densities of shLuc (circle; n=7 for metformin; n=4 for IAA94) and shCLIC1 (squares; n=6 for metformin; n=4 for IAA94). Data are expressed as mean ± sem cells. F and G) Down-regulation of CLIC1 expression slightly affects basal cell proliferation (−14% at 48 hours *vs*. shLuc cells) but significantly reduces the effects of metformin and IAA94 on CSC viability after 48 hours of treatment, evaluated by MTT assay. Results demonstrate that the expression of CLIC1 is required for the antiproliferative activity of metformin in GBM CSCs. Dotted line represents the respective untreated control value for shLuc and shCLIC1 infected cells, taken as 100%. *p<0.05, **p< 0.01 and ***p<0.0001 (t-test).

We knocked-down CLIC1 expression in CSCs by specific shRNA lentiviral infection (shCLIC1), verified by immunocytofluorescence (Fig. 3A) and Western blot (Fig. 3B). Metformin inhibited CLIC1 conductance in wt GBM1 CSCs with an EC_50_ (2.3mM) similar to U87 cells (Fig. 3C). Both IAA94 and metformin reduced CLIC1 current in control (shLuc)-infected CSCs, while CLIC1-silenced CSCs showed lack of ionic flow (Fig. 3D). Current/voltage relationships (Fig. 3E) show, in an average of several cells, a consistent CLIC1 current in shLuc control cells (open circles), while in CLIC1-silenced CSCs the average metformin and IAA94 sensitive currents (open squares) decline by more than 50%. Furthermore, shCLIC1-CSCs show lower metformin (and IAA94) cytotoxicity (Fig. 3F), confirming, at molecular level, that CLIC1 mediates metformin's antiproliferative effects.

**Fig. 4:**
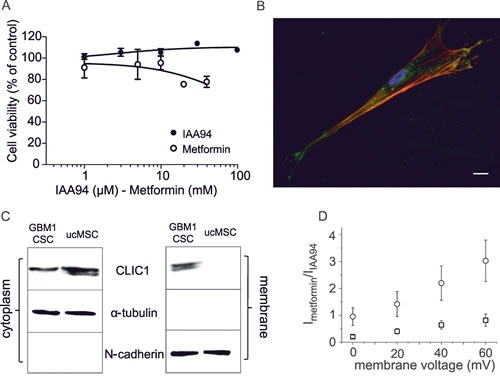
Effects of metformin and IAA94 on umbilical cord-derived mesenchymal stem cells (ucMSCs) viability A) Metformin (1-40mM) and IAA94 (1-100μM) for 48 hours failed to reduce ucMSCs viability, measured by MTT assay, performed in quadruplicate (n=3). B) CLIC1 expression in ucMSCs measured by immunofluorescence. Phalloidin staining (red) highlights cell shape, while indirect immunofluorescence identifies CLIC1 (green). C) Differential CLIC1 expression in ucMSCs and GBM1 CSCs evaluated by Western blotting, in cytosolic and plasma membrane fractions. N-cadherin and α-tubulin expression, used to normalize for membrane and cytosolic proteins, respectively, confirmed the lack of significant protein contamination between cell fractions. Representative blots are reported (n=2). D) IAA94-sensitive current in ucMSCs and GBM1 CSCs. The plot shows the current/voltage relationship of perforated patch whole-cell experiments. IAA94-sensitive current measurement demonstrated that ucMSCs (squares) have negligible CLIC1 membrane current, compared to CSCs (circles).

The specificity of metformin antiproliferative effects towards CSCs [17, 18] was showed demonstrating that metformin (and IAA94) did not affect human umbilical cord-derived mesenchymal stem cell (ucMSC) viability (Fig. 4A), in agreement with previous studies in which metformin was reported to exert a trophic activity for adult stem cells [40]. Differently from GBM CSCs, CLIC1 was not detected in the ucMSC membrane compartment, where it acts as ion channel, but was confined as inactive protein within the cytosol (Fig. 4B and C), thus CLIC1 current was negligible (Fig. 4D). These results suggest that metformin inhibits proliferation only in cells in which CLIC1 functioning is required for proliferation (*i.e.* GBM CSCs) [32], but it spares normal stem cells although these cells retain CLIC1 expression.

### Inhibition of G1-S transition as metformin's antiproliferative mechanism

**Fig. 5:**
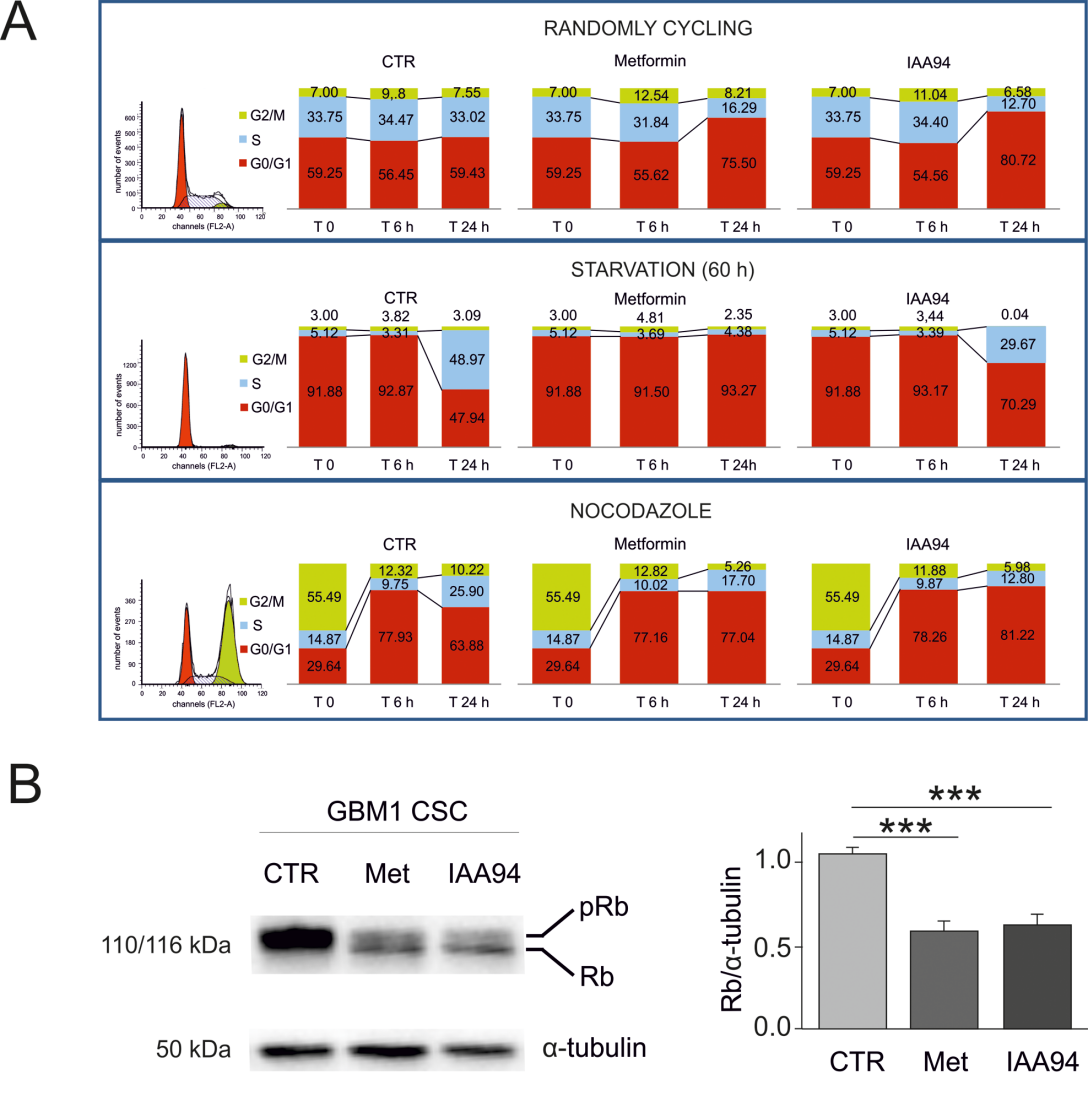
Metformin causes GBM CSC arrest in the G1 phase of the cell cycle A) GBM1-derived CSCs were evaluated by FACS analysis for cell cycle progression after propidium iodide staining (n=2). Control, metformin (10 mM)- or IAA94 (100 μM)-treated cells were analysed after 6 and 24 hours without synchronisation (randomly cycling, upper panels), after G0/G1 arrest (induced by 60 hours of growth factor deprivation, middle panels) or after blockade induced by nocodazole/cytochalasin B treatment (overnight incubation with 50 ng/mL nocodazole + 25 ng/mL cytochalasin B, lower panels). At experimental time 0 (depicted in the original histograms from the FACS on the left, and in the first superimposed bars), randomly cycling cells show the following cell cycle distribution: G0/G1= 59.25%; S=33.75%; G2/M=7%; starvation induced a clear G0/G1 synchronisation (91.88% of the cells, with only 5.12 and 3% in phase S and G2/M, respectively); nocodazole treatment caused cell accumulation in G2/M (55.49%) with 29.64% of the cells in G0/G1 and 14.87% in S phase. Cells were subsequently shifted into standard growth factor-containing CSC medium and allowed to grow for 6 or 24 hours. Treatment with both metformin and IAA94 caused a strong reduction of cell cycle progression that was mainly due to the blockade of G1-S transition. B) Western blot analysis of retinoblastoma (Rb) protein in metformin- and IAA94-treated CSCs. Metformin (10 mM) and IAA94 (100 μM) affects Rb protein expression and phosphorylation status in GBM1 CSCs. Treatment with either drug not only drastically reduced Rb protein content, but also led to a higher prevalence of non-phosphorylated protein in treated cell extracts compared to control conditions. Thus, metformin- and IAA94-induced cell cycle arrest correlates with a reduction of Rb content and a shift towards the non-phosphorylated form. Representative Western blot is reported in the left panel, and the quantification by densitometric analysis of two replica experiments is depicted in the right panel (***p<0.001, t-test).

CLIC1 translocates to the membrane following an increase in cytosolic oxidative state [35, 41, 42] corresponding to a peak of ROS during G1-S transition [43]. Thus, CSCs were synchronized by growth factor starvation (60 hours), to accumulate cells in G0/G1, and nocodazole/cytochalasin B treatment, to freeze cells in G2/M, and cell cycle evaluated by FACS. After the relief of cell cycle arrest and administration of metformin, IAA94, or vehicle (Fig. 5A), randomly cycling cells showed a partial arrest in G1, 24 hours after metformin or IAA94 treatment, consistent with DNA synthesis experiments (Fig. 2E and F). Release of G0/G1 synchronized cells (about 92% of cells in G0/G1) allowed a synchronous re-entry in the cell cycle, mostly evident after 24 hours, that was abolished in the presence of metformin or IAA94. Finally, cells released from nocodazole treatment (55% of cells in G2/M) after 6 hours progressed to G1 in the presence of metformin and IAA94, similarly to untreated cells. However, after 24 hours, while untreated cells continued to S phase, almost 80% of metformin/IAA94-treated cells were blocked in G1 (Fig. 5A). The G1-S transition is therefore the target of both metformin and IAA94's antiproliferative effects. Consistently, 24 hour treatment with metformin (or IAA94) of randomly cycling GBM CSCs, decreased both retinoblastoma protein (Rb) content and its phosphorylation status (Fig. 5B).

### Metformin inhibition of CLIC1 is use-dependent and it is lost in CLIC1 mutants lacking Arg29 within the channel pore

**Fig. 6:**
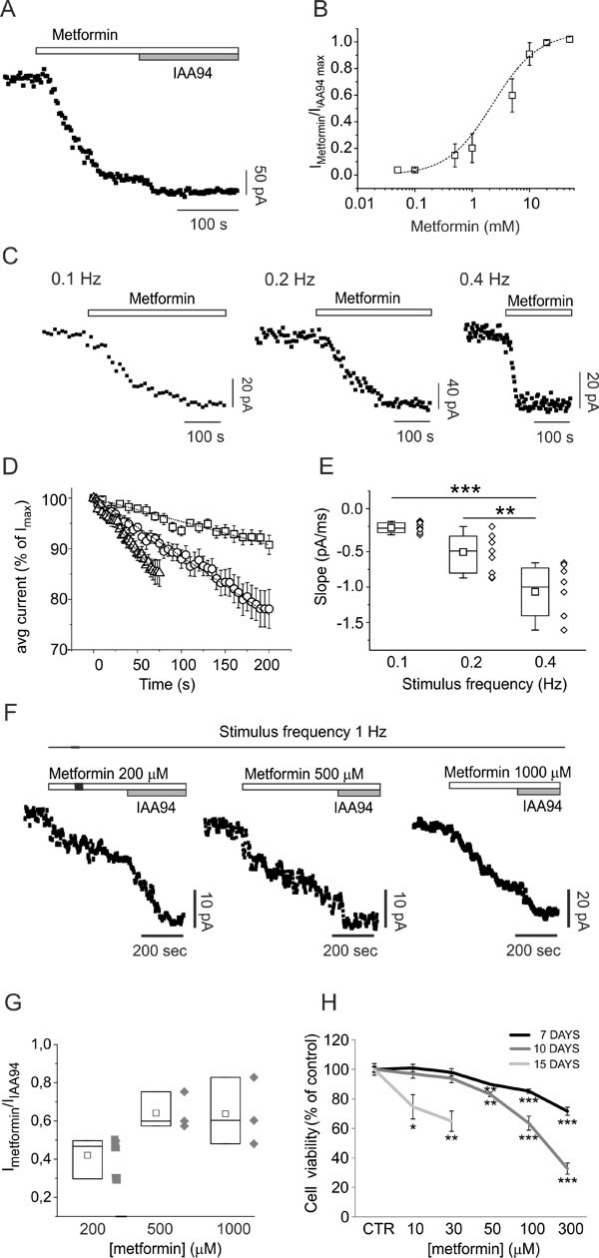
CLIC1 current inhibition by metformin is use-dependent A) Time-course of whole-cell currents in CHO cells transfected with human wt CLIC1, and stimulated every 5 seconds with 800 ms, 100 mV test potential from −40 mV holding voltage. Each point represents the average current of the last 100 ms of a single current trace. Once the current amplitude reached a constant value, the cell was challenged with 10mM metformin. At a new steady-state the cell was perfused with 100μM IAA94 that was used to completely block CLIC1 conductance. B) Dose-response curve built perfusing cells with different metformin concentrations (0.05-50 mM) followed by IAA94 (100μM). Each point is the average result of 5 measurements. C) Examples of metformin (10mM) inhibition kinetics in three different experiments in which cells were stimulated with voltage steps delivered every 10, 5 or 2.5 seconds. D-E) Negative slope portions of several experiments, as in panel C, at different stimulation frequencies were normalised as percentages of the maximum current value of each trial. Average inhibition time-courses at stimulation frequencies 0.1 (squares; n=7), 0.2 (circles; n=9), and 0.4 Hz (triangles; n=8) were plotted and fitted with a linear regression function (D). Calculated slopes of the average inhibition of time-course currents (0.1 Hz= −0.231±0.01; 0.2 Hz= −0.577±0.01; 0.4 Hz= −1.052±0.02) are coincident with the average result obtained by plotting single-experiment metformin inhibitory slopes in a box-plot (E). Higher frequency stimulation induces faster inhibition of the current. The difference between 0.1 and 0.2 Hz is not statistically significant (p=0.12), but stimuli at 0.4 Hz are significantly different from slower stimulation frequencies (**p <0.001, ***p <0.0001, one-way ANOVA, followed by Tukey test). F) Representative metformin-sensitive membrane current time-course of perforate whole-cell patch clamp experiment in GBM CSCs, at high frequency stimulation. Voltage step (100 mV, 400 ms) from −40 to +60 mV of membrane potential was delivered every second (1 Hz). Once the membrane current amplitude was stabilized, metformin was perfused during continuous stimulation. In all cases membrane current decreases but was not further inhibited by IAA94 (100μM). G) Ratio between metformin and IAA94-sensitive current in the different conditions reported (n=3). H) CSC viability measured by MTT assay after 7 (black line), 10 (dark grey) or 15 (light grey) days of metformin treatment (n=2; *p<0.05, **p< 0.01 and ***p<0.0001, t-test *vs*. vehicle-treated cultures).

The direct metformin interaction with CLIC1 was tested in CHO cells transfected with *wild type* (wt) and mutant CLIC1. In CLIC1 wt-transfected cells, whole-cell electrophysiology recordings show a slow (few minutes) metformin inhibition of chloride current (Fig. 6A and B), similar to what observed in GBM cells. This kinetics was explained as an activity-dependent inhibition of CLIC1 current due to a direct interaction with the channel. Indeed, CLIC1 inhibition by metformin was dependent on the frequency of stimulation (Fig. 6C, D and F), suggesting that metformin binds CLIC1 only in the open state.

In this and previous reports, metformin antitumor effects occur at concentrations in the millimolar range (1-50mM) [8, 9, 25, 44]. Such high concentrations however, although referred as “oncobiguanides” treatments [45], were often questioned as far as the *in vivo* significance of these effects. Nevertheless, the lack of toxicity in differentiated GBM cells and in ucMSCs, as demonstrated in our study, argues against a non-specific *in vitro* effect. In addition, prolonged treatment with equimolar concentration of arginine did not reduce CSC viability (Fig. S5), indirectly confirming that the observed effects are specific for metformin.

Importantly, our data provide a mechanistic interpretation for the apparent incongruence between *in vitro* effective concentrations and *in vivo* achievable drug levels. According to our results (Fig. 4), metformin acts only when CLIC1 is active in the membrane. Given the short timing of channel activity, the limited number of CLIC1 molecules present on the membrane, and the relatively low open channel probability [41], the chance to find an open CLIC1 channel in the membrane during the G1-S transition is very low. In acute electrophysiology experiments or short-time cell viability experiments, being CLIC1 inhibition by metformin practically irreversible, only high concentrations of metformin (*i.e.* 10mM) can inhibit a significant percentage of active channels to obtain inhibition of cell proliferation. Thus, the antiproliferative effects are not only strongly dependent on metformin concentrations but also on the duration of treatment, considering that in clinical trials prolonged (weeks or months) *in vivo* treatments are performed [46].

Accordingly, high frequency stimulation (in which more open channels are simultaneously available to metformin) or longer exposure to the drug (in which a prolonged inhibition progressively blocks the activity of all CLIC1 pools that reach cell membrane during cell cycle progression) induced significant effects using lower *in vitro* metformin concentrations.

Metformin (200-1000μM) significantly inhibited CSC CLIC1 current during high frequency stimulation (1Hz, Fig. 6F and G). Furthermore, in accordance with data using U87 cells (Fig. 1G), prolonged treatment (up to 15 days) with low doses of metformin (10-300μM) significantly reduced CSC viability (Fig. 6H), displaying a precise inverse relationship between treatment duration and the concentration required to reduce CSC viability (*i.e.* 10-30μM metformin are sufficient to elicit an effect after 15 days, while 50 and 100μM required 10 and 7 days).

Experimental data suggest that the pore formed by CLIC1 has a single, 22 amino acids, transmembrane (TM) domain [29, 30, 35, 42, 47]. FRET data [42, 47] showed that TM segment forms an amphipathic α-helix where the only charged residues are Arg29 and Lys37. CLIC1 channel conformation is likely to be an oligomer of 7±1 monomers [42], similar to viroporin, formed by oligomerization of a single TM helix [48, 49], in which multiple copies (7±1) of Arg29 and Lys37 line the anion path. Arg29 is likely to be near the mouth of the pore at the cell surface, while Lys37 is in the middle of the TM region. Consistent with this model, R29A and K37A mutations alter CLIC1 biophysical parameters [41]: Arg29 destabilizes the closed state of the channel and the R29A mutation results in a closed channel that only opens at positive membrane potential (away from the chloride reversal potential) [41]. Arg29 and/or Lys37 may therefore form part of the metformin binding site [35, 42]. Thus, we substituted each of them with alanine (R29A and K37A) [41], and measured metformin's effects on CLIC1 currents in transfected CHO cells.

**Fig. 7:**
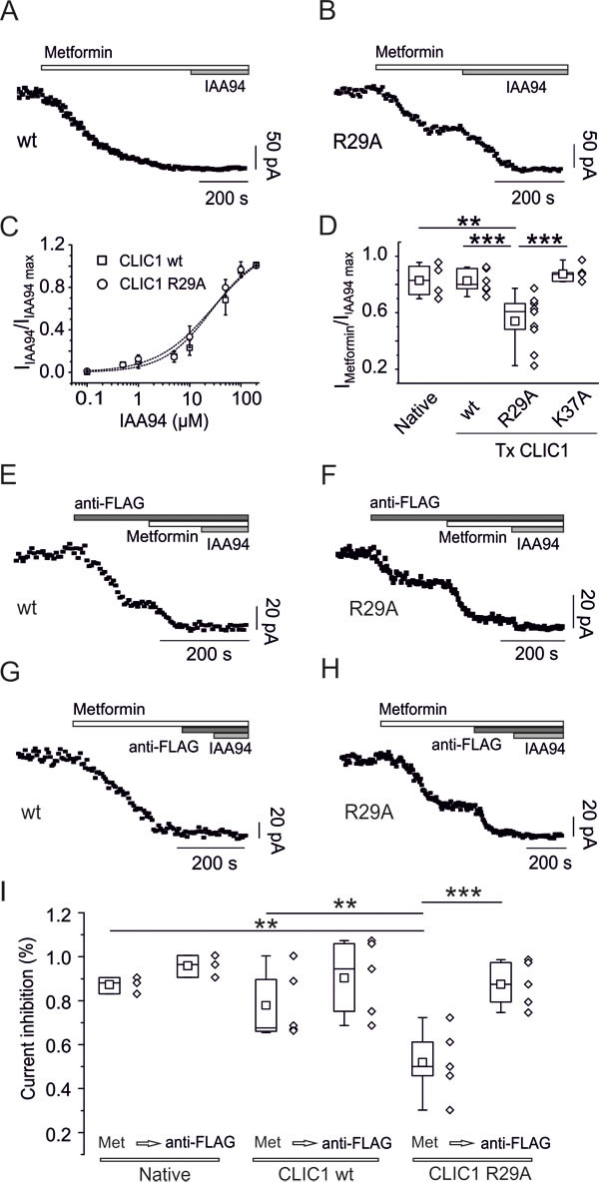
CLIC1 point mutation impairs metformin effects A) Whole-cell current time-course of CHO cells transfected with CLIC1-wt, stimulated every 5 seconds with 800 ms, 100 mV test potential from −40 mV holding voltage. Metformin (10mM) inhibits membrane current, which was not further decreased by IAA94 (100μM). B) Similar experiments as in A, using CHO cells transfected with CLIC1 R29A show that metformin (10mM) achieves only a partial block of IAA94 (100μM)-sensitive current. C) Representative dose-response curves of IAA94-dependent current inhibition demonstrate that both CLIC1 constructs transfected in CHO cells have a similar EC_50_: 27.3 μM for CLIC1-wt (squares, n=4, data expressed as mean±s.e.m.), and 29.2 μM for CLIC1-R29A (circles, n=4). Data are reported as inhibition of IAA94-sensitive current, over maximal inhibition. D) Box-plot quantifying metformin-insensitive portion of IAA94-sensitive current. Ratio between metformin (10mM) and IAA94 (100μM) is reported for native CHO cells (n=4, mean 0.83±0.06), cells transfected (Tx) with CLIC1-wt (n=8, mean 0.83±0.03), CLIC1-R29A (n=9, mean 0.54±0.06) and CLIC1 K37A (n=5, mean 0.87±0.028). Only the trials with CLIC1-R29A produced a value significantly different from the others (**p<0.01 *vs*. native CHO; ***p<0.001 *vs*. CLIC1 wt and K37A, one-way ANOVA, followed by Tukey test). E-F) Currents from transfected CLIC1 constructs are functionally identified using anti-FLAG antibody acting as current blocker (see text). Both CLIC1 wt- (E) and R29A-transfected cells (F) show partial inhibition of membrane current by anti-FLAG antibody while the remaining current, representing endogenous CLIC1, was blocked by both metformin (10mM) and IAA94 (100μM). G-H) Metformin induces a total membrane current knockdown in CLIC1 wt-transfected cells, without a further decrease caused by anti-FLAG antibody or IAA94 (G). In R29A-expressing cells, metformin only partially blocks the current, which is fully inhibited by anti-FLAG antibody or IAA94 (H). I) Summary box-plots demonstrating that in CLIC1 R29A-transfected cells a consistent part of the current is insensitive to metformin (Met). Mean I_met_/I_IAA94_ ratios are 0.84±0.02 (n=3), 0.76±0.08 (n=5), 0.52±0.07 (n=5) for native, transfected wt, and R29A mutant CLIC1, respectively. The current ratio in R29A transfected cells is statistically different from both native and CLIC1 wt-transfected cells. Further perfusion with anti-FLAG antibody equalizes IAA94-sensitive current inhibition showing a mean I_anti-FLAG_/I_IAA94_ ratio of 0.96±0.03 (n=3), 0.9±0.08 (n=5), 0.87±0.05 (n=5) for native, wt and R29A CLIC1, respectively. In R29A-transfected cells metformin and anti-FLAG current inhibitions are statistically different. **p<0.01 and ***p<0.001 (one way ANOVA followed by Tukey test).

Metformin significantly inhibited membrane current in CLIC1 wt and K37A-transfected cells (Fig. 7A and D), but only partially in R29A-transfected cells (Fig. 7B). Conversely, similar IAA94 dose-response curves were observed in CLIC1 wt- and R29A-expressing cells (Fig. 7C).

CLIC1 current in transfected CHO cells derives from a mixture of endogenous and transfected proteins. The current induced by the transfected protein is easily distinguishable from the endogenous one, since transfected channels are FLAG-tagged, and anti-FLAG antibody acts as channel blocker [29, 31]. Both wt- and R29A-expressing cells showed part of IAA94-sensitive current blocked by anti-FLAG antibody (Fig. 7E and F). Metformin completely abolished IAA94-sensitive membrane current in CLIC1-wt cells, with no further decrease observed in presence of the anti-FLAG antibody (Fig. 7G). Conversely, in R29A-transfected cells, after partial current inhibition by metformin, anti-FLAG antibody further decreased membrane current (Fig. 7H). Average results (reported in Fig. 7I) support R29 requirement for metformin inhibition of CLIC1.

**Fig. 8:**
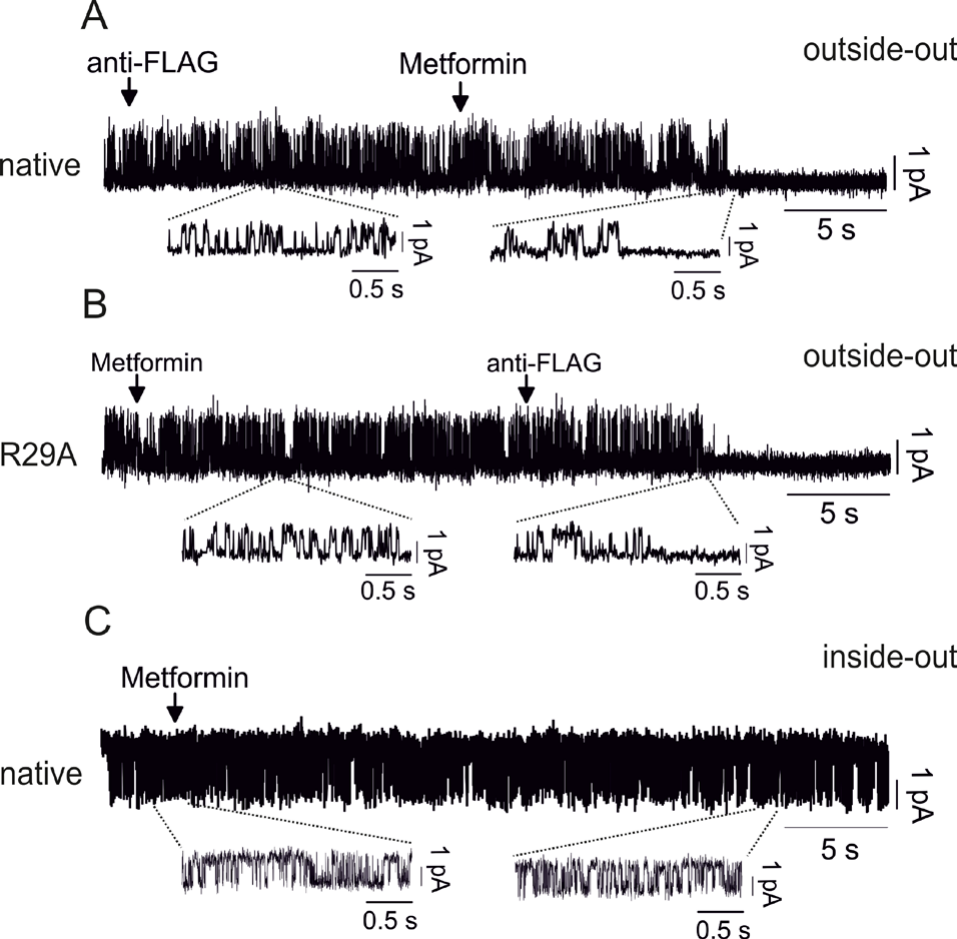
Molecular characterization of CLIC1 activity modulation by metformin A) Outside-out single channel experiments in native CHO cells show that anti-FLAG antibody does not inhibit CLIC1 current, differently from metformin (10mM) that closes the channel (single wt channel in control conditions P_open_= 0.2±0.01, n=3 total of 6 minutes continuous recording). B) CLIC1 R29A, transfected in CHO cells, is insensitive to metformin, but it is promptly blocked by anti-FLAG antibody (single R29A channel in control conditions P_open_=0.21±0.008, n=3 total of 7 minutes continuous recording). C) Inside-out single channel experiments show that metformin fails to inhibit CLIC1 channel opening after more than 30 seconds of continuous recording (single wt channel in control conditions P_open_= 0.26±0.02, n=3 total of 10 minutes continuous recording).

Outside-out single-channel experiments further confirmed that metformin directly acts on CLIC1 wt from the external side (Fig. 8A) but failed to shut-down single channels in CLIC1 R29A-transfected cells (Fig. 8B). Accordingly, metformin was unable to inhibit CLIC1 single channel current in inside-out trials (Fig. 8C).

## DISCUSSION

Drug repositioning represents a growing field in pharmacology research, opening in the last few years, unexpected, yet promising, novel approaches in particular for anti-cancer purposes [6, 50-53]

The crucial message coming out from our study is the identification of a molecular target for the antiproliferative activity of metformin in human GBM CSCs. Recently, several studies reported the *in vitro* and *in vivo* efficacy of metformin as antitumoral agent for most human tumors, including GBM [17, 18], showing its efficacy in reducing proliferation, survival, clonogenicity and *in vivo* tumorigenicity of CSC subpopulations. While these studies opened the way to ongoing clinical trials [2, 54], in most cases, they did not address two central issues: (i) the molecular mechanism by which metformin reduces tumor development and growth (considering that, at odds with the initial reports, most studies are now showing that AMPK activation is not the main player in this effect [19-21]), and (ii) the mechanisms for metformin selectivity towards cancer cells, a rather unique characteristic for antitumoral drugs, but also demonstrated by the absence of significant toxicity of metformin when chronically used in diabetic patients.

The goal of our study was to provide answers to these questions, using CSC cultures, a reliable *in vitro* model of human GBM [38]. We propose CLIC1, a transiently active chloride channel previously identified as a requirement for GBM growth *in vivo* and *in vitro* [32], to be the molecular target of metformin activity. We show that metformin targets CLIC1 “functional expression” only when the protein translocates to the plasma-membrane where it acts as chloride conductive pore to allow the G1-S transition of the cells. Conversely, neither metformin nor IAA94 (a selective CLIC1 inhibitor) reduce proliferation of differentiated GBM cells or ucMSCs, in which CLIC1 is similarly expressed but confined to the cytosol in an inactive conformation. Thus, the different activity and availability of CLIC1 as active chloride channel in normal and tumoral cells, dictates the strict selectivity of metformin towards CSCs. The specificity of metformin's effects is corroborated by the observation that in diabetic patients and in experimental studies, chronic metformin treatment is not toxic to normal stem cells [55, 56], in contrast with mTOR inhibitors [55]. The observations that CLIC1 chloride current is essential for human GBM CSC proliferation [32] and that metformin preferentially affects CSC viability [17] support this assumption.

**Fig. 9:**
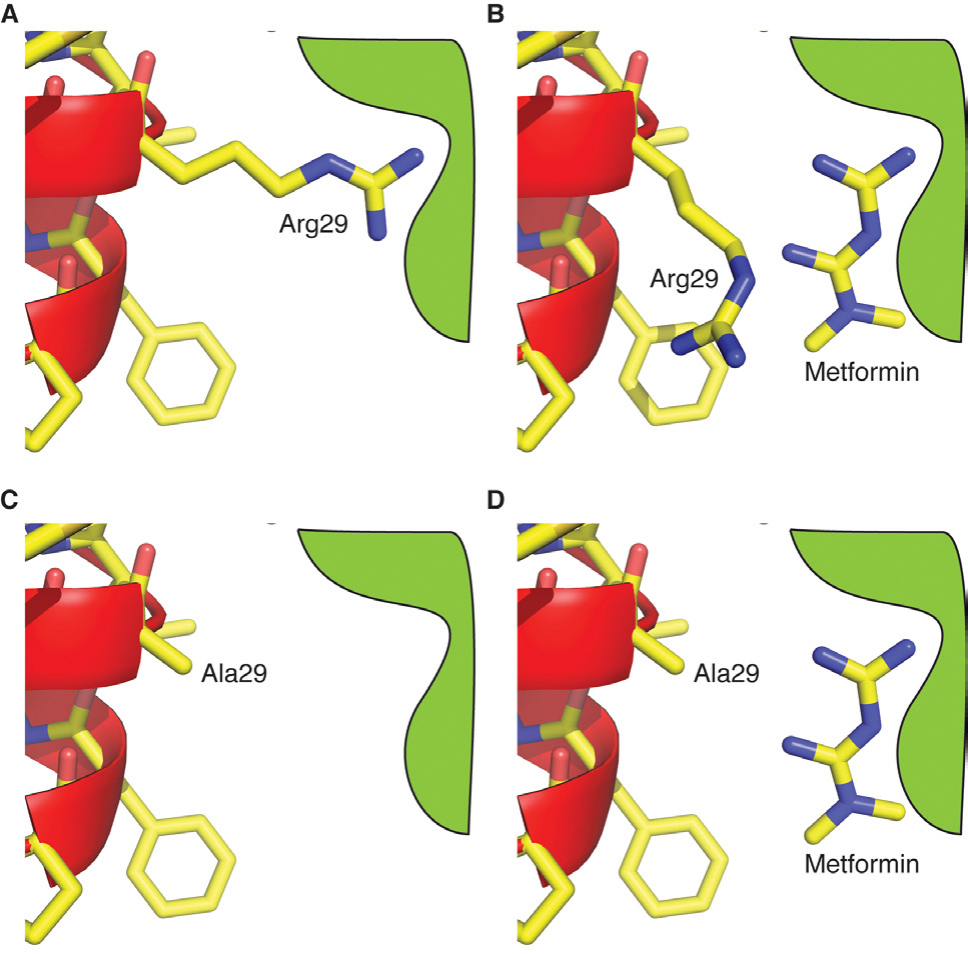
Modelling of metformin-CLIC1 interaction Schematic representation showing a possible mechanism of metformin's inhibition of CLIC1 wt, while it has no effect on R29A mutant. A) In the closed state of CLIC1 wt, the side chain of Arg29 makes an interaction that destabilizes the closed state. This facilitates the opening of the channel near the chloride reversal potential. B) Metformin displaces the guanidinium moiety of Arg29, stabilizing the closed state. In addition, the displaced Arg29 side chain may obstruct the channel pore. C) In the R29A mutant, the closed state is stable near the chloride reversal potential, as there is no Arg29 side chain to make the destabilizing interaction as it is replaced by Ala29. D) Metformin is free to bind to the guanidinium site in R29A, as it is unoccupied. It has no effect on channel opening at high membrane potentials.

The transient functional activation of CLIC1 during mitosis in CSCs allows metformin to act as a selective CLIC1 inhibitor to slow-down cell cycle progression. Importantly, metformin time-dependent efficacy highlighted in this study might reconcile the different concentrations required to affect tumor proliferation *in vitro* and *in vivo*, with prolonged *in vivo* treatments likely being effective also with lower, clinically reachable, metformin concentrations. Moreover, the experiments using CLIC1 mutants, here reported, provided clues for the mechanism of the metformin-CLIC1 interaction. We demonstrate that metformin interacts with CLIC1 from the external side of the membrane at the amino terminus of the channel, presumably near the side chain of Arg29, which is responsible for destabilizing the closed state of the channel. These data allowed us to build a conceptual model for metformin's action. Arg29 is likely to be near the anion path proximal to the extracellular surface. As it destabilizes the closed state of the channel, it could interact with a polar portion of the channel *via* its guanidinium moiety (Fig. 9A). Using its double guanidinium group, metformin may displace the side chain of Arg29 from this polar pocket, stabilizing the closed state and possibly obstructing the channel pore displacing the large arginine side chain (Fig. 9B). Metformin can only access this polar site from the outside of the cell, as the binding site is near to the cell surface, given the position of Arg29 in the TM segment (consistent with the lack of effects in inside-out electrophysiology experiments). In contrast, CLIC1-R29A, lacking Arg29 side chain, displays a more stable closed state, only opening at high membrane potential (Fig. 9C). The polar binding pocket (normally occupied by the guanidinium moiety of Arg29) is exposed and free to bind metformin (Fig. 9D). However, metformin binding has no effect on the electrophysiological properties of the Arg29-lacking mutant, as there is no arginine side chain to displace to cause channel inhibition.

The observation that metformin effects in GBM CSCs may occur through an interaction on CLIC1 Arg29 from the external side on cell membrane (see single channel recordings reported in Fig 8C) suggests that metformin antiproliferative activity in GBM CSCs is, at least partially, independent from its intracellular accumulation. However, comparing the effects of metformin and IAA94 (a “pure” CLIC1 inhibitor) some differences in efficacy and cell sensitivity are apparent in GBM cells (see cell viability and cell cycle data). This discrepancy highlights the possibility that additional cellular effects could be induced by metformin but not by IAA94. In fact, although several of the previously reported effects of metformin in cancer cells could be reconciled with the cell cycle regulation induced by CLIC1 inhibition (i.e. modulation of kinase systems that control proliferation and/or survival [17, 22, 24], or the activation of transcription factors [26, 27]), recent data also reported a direct effect of this drug on complex I and oxidative phosphorylation, which, besides being responsible for its metabolic effects, could also interfere with cell proliferation [54, 57]. In particular, due to the avid glucose consumption as energy source for cancer cells, metformin antiproliferative effects were also reported to be mediated by alteration of cancer cell glucose metabolism via a direct inhibition of hexokinase II and its dissociation from mitochondria, to form an inactive cytosolic complex with metformin [58, 59]. As a consequence, glucose availability may also modulate cancer cell sensitivity to metformin [60, 61]. Thus, both metabolic (glucose metabolism) and transductional (CLIC1 inhibition) effects may account for synergistic antiproliferative effects of metformin.

The intracellular cascade activated by CLIC1 (and inhibited by metformin) responsible for CSC proliferation is still to be elucidated. From previous studies several possibilities could be considered including an oxidative environment generated on a rapid time-scale, which facilitates the progression of mitosis [43], CLIC1 interaction with the cytoskeleton [62] and regulation of cell volume [63], which may ultimately affect cell viability via intracellular kinases [17].

In conclusion, metformin, whose ability to cross the blood-brain barrier was reported [18, 64], could represent a valuable therapeutic tool for GBM, due to its negligible side-effects, easy delivery, and low cost. Future clinical trials are required to definitely demonstrate metformin *in vivo* efficacy, although its time-dependent, low dose efficacy represents a good starting point to translate these results to the clinics. In addition, CLIC1 is a novel pharmacological target for newly designed drugs due to its accessibility from outside the cell and transient appearance in the membrane only during deregulated cell cycle progression.

## MATERIALS AND METHODS

### Reagents and antibodies

Metformin (1,1-dimethylbiguanide-hydrochloride) and IAA94 (indanyloxyacetic acid 94) were from Sigma-Aldrich (Milano, Italy).

Antibodies: anti-CLIC1, Santa Cruz (Dallas, USA); anti-GFAP, anti-Nestin, and anti-N cadherin, Abcam (Cambridge, UK); anti-α-tubulin, anti-β-III tubulin, anti-FLAG M2-Cy3 and anti-FLAG M2, Sigma-Aldrich (Milano, Italy); anti-Olig2 and anti-Sox2, Millipore (Vimodrone, Italy); anti-Rb, BD Biosciences (Milano, Italy); Alexa Fluor 488 anti-mouse and Alexa Fluor 546 anti-phalloidin, Invitrogen (Carlsbad CA, USA); DyLight 459-goat anti-rabbit IgG, Jackson Immunoresearch (West Grove, USA).

### Human GBM CSC and ucMSC cultures

GBM CSC cultures were obtained from 2 females and 1 male patients (67, 40 and 71 years old, respectively) and coded as GBM1, GBM2, GBM3. Post-surgical samples were used after patients' informed consent and Institutional Ethical Committee (IEC) approval. All patients underwent surgery at Neurosurgery Department (IRCCS-AOU San Martino-IST) and had not received therapy prior to the intervention. Specimens were histologically classified as GBM grade IV (WHO classification).

CSCs, isolated as described [65, 66], were grown in stem cell-permissive medium enriched with 10ng/ml human bFGF and 20ng/ml human EGF [39]. Sphere formation occurred within 2 weeks of culture (Fig. S3). CSCs were also grown as monolayer on growth factor-reduced Matrigel coating (BD Biosciences), allowing easier evaluation of viability experiments without affecting stem cell features [65]. Validation of CSC properties of the cultures (*i.e*. self-renewal capacity, stem cell marker expression, multipotency, and tumorigenicity), was performed as described [17](see Fig. S3). Tumorigenicity was assessed in 6-8-week-old NOD-SCID mice (Charles River, Calco, Italy) (Fig. S3D) after IRCCS-AOU San Martino-IST (Genova, Italy) IACUC approval.

Human umbilical cords were obtained after caesarean section at Obstetrics and Gynaecology Department of International Evangelical Hospital (Genova, Italy), following informed consent and approval by IEC. After vessel removal, cords were treated with collagenase (0.5μg/ml) to expose Wharton jelly and obtain single cells. Cells were grown in MesenPRO RS basal medium+Supplement (Life Technologies, Milano, Italy) after flow cytometry phenotypical characterization (MSC Phenotyping Kit, Miltenyi Biotec). Briefly, more than 95% ucMSCs were negative for hematopoietic antigens (CD45, CD34, CD14) and MHC class-II, and showed expression of CD73, CD105, CD90, CD29, and MHC class I. After incubation in selective media, ucMSCs differentiate into osteocytic, chondrocytic, and adipocytic lineages.

### Cell lines and transfections

U87-MG and Chinese Hamster Ovary-K1 (CHO) were from Interlab Cell Line Collection (ICLC, Genova, Italy) and grown following standard conditions. Both cell lines were validated by ICLC. CLIC1 wt, R29A, and K37A mutants, tagged with FLAG peptide, were stably expressed in CHO cells using FuGENE Reagent (Roche, Basel, Switzerland) [30].

### Electrophysiology

Patch electrodes (GB150F-8P with filament, Science Products) were pulled from hard borosilicate glass on a Brown-Flaming P-87 puller (Sutter Instruments, Novato, USA) and fire-polished to a tip diameter of 1-1.5μm and an electrical resistance of 5-7 MΩ. Patch-clamp electrophysiology was performed in whole cell, perforated-patch whole cell, and single channel outside-out and inside-out configurations, as reported [32]. Single channel experiments were performed as previously described [41].

### Cell proliferation assays

Mitochondrial function, as index of cell viability, was evaluated by measuring the reduction of 3-(4,5-dimethylthiazol-2-yl)-2,5-diphenyltetrazolium bromide (MTT, Sigma-Aldrich) as reported [67].

DNA synthesis was evaluated by the measurement of 5-bromo-2′-deoxyuridine (BrdU) incorporation in replicating DNA (Cell proliferation ELISA, Roche), following the manufacturer's instructions.

For cell cycle analysis, cells were synchronised by growth factor starvation (60 hours), or nocodazole treatment (overnight incubation with nocodazole (50ng/mL)-cytochalasin B (25ng/mL), in DMEM). Randomly cycling and synchronised cells were treated in stem cell-permissive medium [39], and cell cycle analysis performed by flow cytometry (FACScalibur, BD Bioscience) with propidium iodide DNA staining [68].

### Gene silencing

Short hairpins mRNA specific for human CLIC1 (5′-GATGATGAGGAGATCGAGCTC-3′) and firefly luciferase (5′-CGTACGCGGAATACTTCGA-3′) were cloned into the XhoI/HpaI sites of the pLentiLox 3.7 lentiviral vector [32] and stably expressed in GBM CSCs.

### Western blot

Whole cell lysates were obtained using a buffer containing 1% Igepal, 20 mM Tris-HCl, pH 8, 137 mM NaCl, 10% glycerol, 2 mM EDTA, 1 mM phenylmethylsulfonyl fluoride, 1 mM sodium orthovanadate, 10 mM NaF (all from Sigma-Aldrich), and the “Complete” protease inhibitor mixture (Roche) for 10 min at 4°C and proteins quantified using the Bradford assay (Bio-Rad Laboratories, Segrate MI, Italy). Membrane/cytoplasm fractions were obtained using the “Membrane protein extraction kit” (Thermo Scientific), following the manufacturer's instructions. For Western blot experiments, proteins (20-60 μg) were resuspended in Laemmli buffer (2% SDS, 62.5 mM Tris, pH 6.8, 0.01% bromophenol blue, 1.43 mM β-mercaptoethanol, 0.1% glycerol), subjected to 10-12.5% SDS-PAGE, electroblotted onto polyvinylidene difluoride membrane (Bio-Rad Laboratories) and probed with specific antibodies. Immunocomplexes were detected using a chemiluminescence system (Immobilon, Millipore), and the ChemiDoc XRS apparatus (Bio-Rad Laboratories), as reported [69].

### Statistical Analysis

All experiments were repeated at least twice (quantitative data were collected from experiments performed in triplicate or quadruplicate), and expressed as mean ± s.e.m. Statistical analyses and EC_50_ values, calculated using nonlinear regression curve fit analysis selecting the log(drug) *vs*. response-variable slope (four parameters) equation were done using Prism version 5.02 (GraphPad San Diego CA, USA). Statistical significance between groups was assessed by t-test (unpaired, two-tailed) or one-way ANOVA followed by Dunnett's multiple comparison post-test or Tukey tests. Statistical significance was established at p values <0.05.

## SUPPLEMENTARY MATERIAL FIGURES


