# Supplementary material for: Metformin repositioning as antitumoral agent: selective antiproliferative effects in human glioblastoma stem cells, via inhibition of CLIC1-mediated ion current
Source: Oncotarget. 2014 Oct 21;5(22):11252–68. doi: 10.18632/oncotarget.2617 (PMC4294381; doi:10.18632/oncotarget.2617)
Supplement: Supplementary file 1 [file oncotarget-05-11252-s001.pdf]

Metformin repositioning as antitumoral agent: selective antiproliferative effects in human glioblastoma stem cells, via inhibition of CLIC1-mediated ion current

Supplementary Material

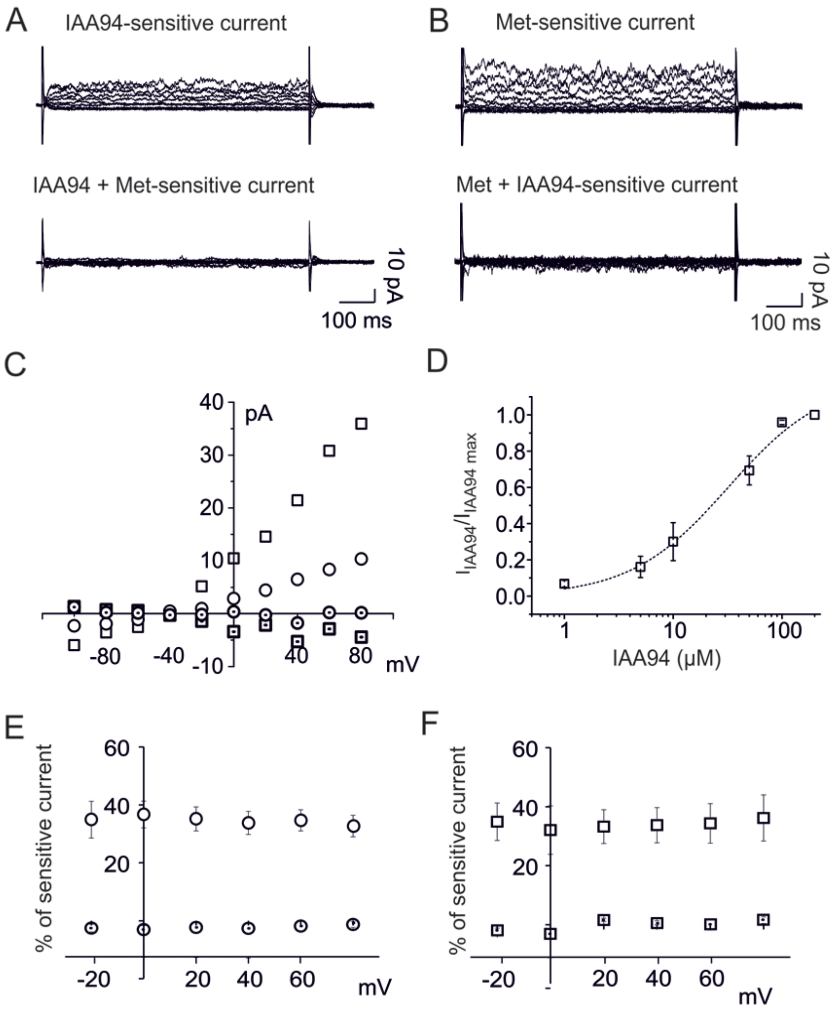

Supplementary Figure 1: **Quantification of metformin-sensitive ion current in U87 cells.**

A and B) Representative traces of metformin- and IAA94-sensitive currents recorded in whole-cell configuration elicited by different 800 ms voltage steps (from -80 mV to 80 mV). IAA94 and metformin were perfused on the same single cell in this sequence (A) and in the reverse order (B).

C) Current-voltage relationships for the corresponding experiments depicted in A and B, exposing the cells to metformin (squares) or IAA94 (circles); the application of either drug after the other results in a null current (dotted squares and dotted circles).

D) Metformin sensitivity was evaluated using IAA94 (100 µM) as a total CLIC1 current inhibitor, as shown by the dose/response curve (EC_{50} = 32 µM, n=4).

E and F) The average results of n=4 and n=5 experiments where IAA94 (circles) (E) and metformin (squares) (F) were used alternately as a first CLIC1 channel inhibitor followed by the other (dotted circles and dotted squares). The average of several experiments shows that metformin and IAA94 share the same target.

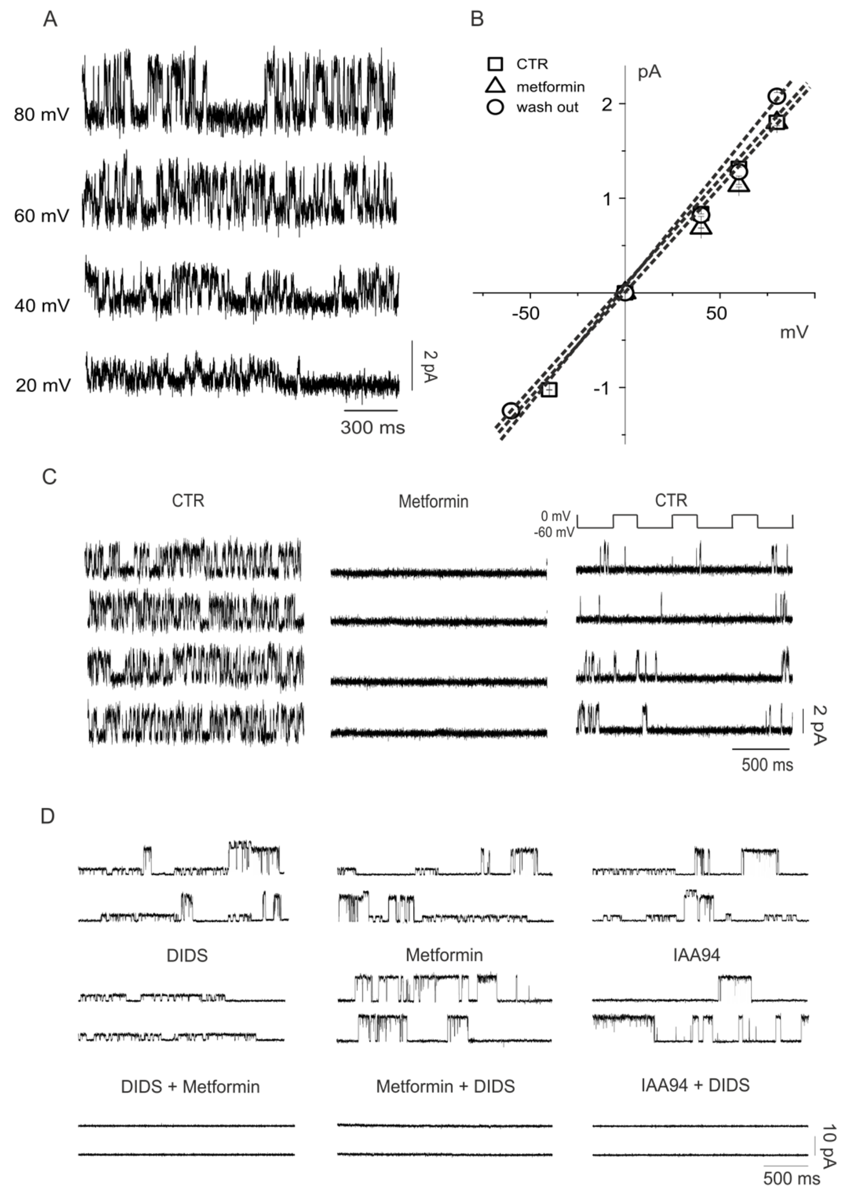

Supplementary Figure 2: **CLIC1 single channel properties and metformin activity in U87 cells.**

A) Outside-out single-channel patch-clamp experiments show CLIC1 channel openings at different test voltages from 0 mV holding potential. (single channel in control conditions P_{open}= 0.23- 0.005, n=3 total of 4 minutes continuous recording at 60 mV membrane potential).

B) Three different single-channel current/voltage relationships relative to the experiment in A (squares) and to the rescue experiment in C (circles before and triangles after wash out). Single channel conductance is 21.95 - 1.33 pS for control (A), 25.57 - 0.45 and 23.61 - 1.72 pS for the experiment in (C) before metformin and after wash out, respectively (n = 3 in each condition).

C) CLIC1 single channel opening (left) was completely abolished by 2 minutes of metformin (10 mM) perfusion (middle). However, following 3 x 1 Hz stimulation from -60 to 0 mV for 2 minutes, low probability transition of the channel from the closed to the open state was observed (right). Although the rescue single-channel shows a low open probability, the unitary conductance observed is similar to that in controls (B). Traces representative of n=3 experiments.

D) Metformin current inhibition is selective for the CLIC1 ion channel. It is not unusual, in these experimental conditions, to observe two different single channel openings in outside-out recording. In the traces reported, the largest channel shows a conductance of 205.11 - 0.87 pS. This particular conductance is blocked by 200 µM DIDS (left) while it is insensitive to 10 mM metformin (middle) or 100 µM IAA94 (right), that, conversely, abrogates the small conductance channel. Simultaneous perfusion with DIDS and metformin or IAA94, completely blocks all of the recorded current (lower traces).

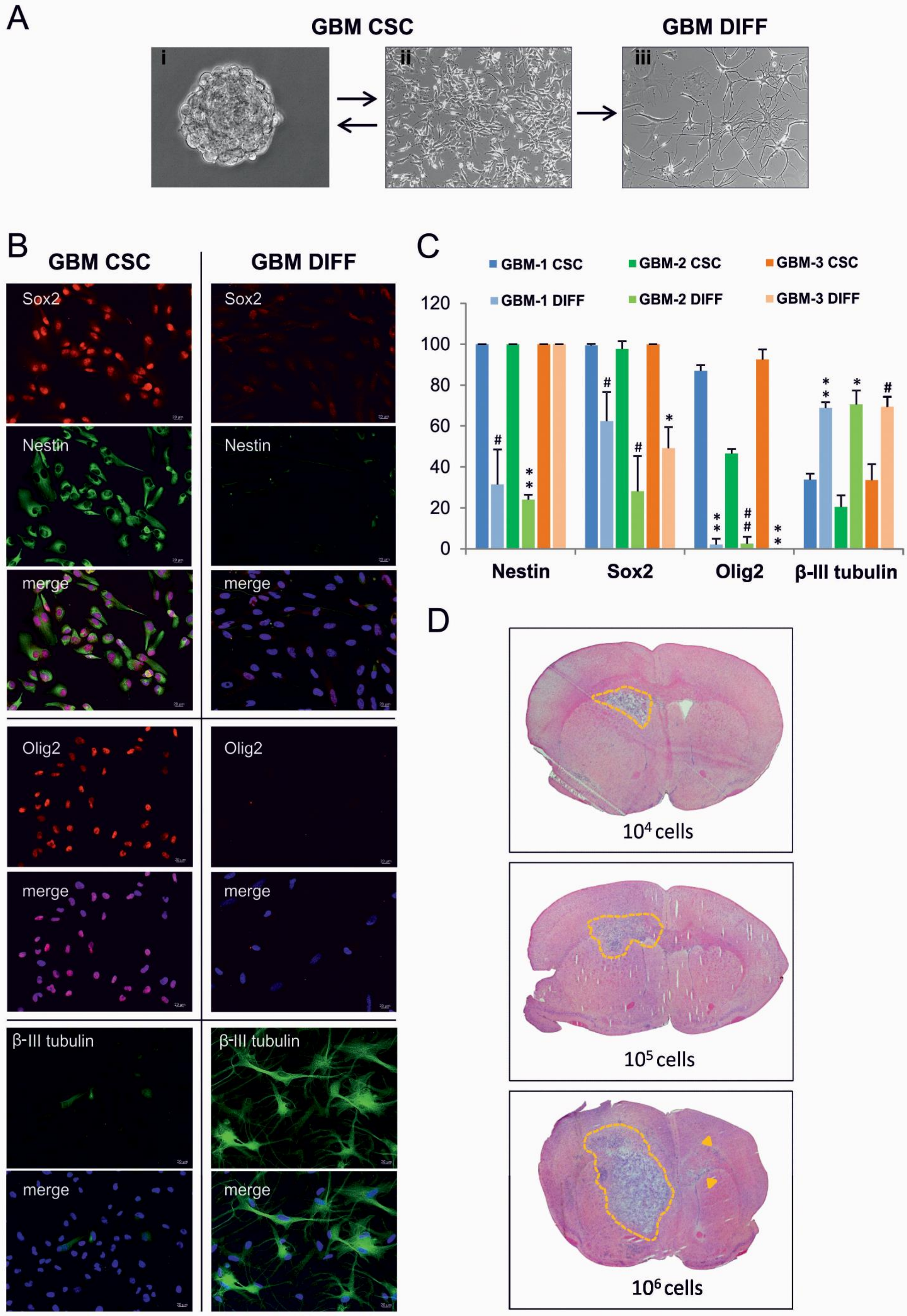
A
GBM CSC
GBM DIFF
i
ii
iii
B
GBM CSC
GBM DIFF
Sox2
Nestin
merge
Olig2
merge
β-III tubulin
merge
C
GBM-1 CSC
GBM-2 CSC
GBM-3 CSC
GBM-1 DIFF
GBM-2 DIFF
GBM-3 DIFF
120
100
80
60
40
20
0
Nestin
Sox2
Olig2
β-III tubulin
D
10^4 cells
10^5 cells
10^6 cells

Figure S3: **Human glioblastoma stem cell characterisation.**

CSCs were isolated from post-surgical specimens of three GBMs (WHO IV), as reported [65]. Cells were grown in stem cell-permissive medium or shifted into growth factor-free and FBS-containing medium to induce cell differentiation. CSC cultures grew as floating spheroids or as monolayers on matrigel, a culture condition that does not affect CSC biological properties such as tumorigenicity and stemness marker expression [17,65].

A) Representative micrographs of GBM1 cells growing as spheroids (i), a monolayer (ii), or after differentiation (iii).

B) Representative differential expression of stem and differentiation markers in GBM1 cells grown in CSC-permissive medium or after FCS-dependent differentiation. A dramatic down-regulation of the stem cell markers Sox2, Nestin, and Olig2 was observed in differentiated cells compared to CSCs. Conversely, differentiated GBM cells overexpress the neuronal marker βIII tubulin, which is detectable in only a few CSCs. Nuclei are counterstained with DAPI.

C) Quantification of the percentage of Sox2-, Nestin-, Olig2-, and βIII tubulin-positive CSCs and differentiated cells from GBM1, GBM2 and GBM3. Almost all CSCs from the three tumours express stemness markers (Sox2, Nestin, Olig2) and at low percentages βIII tubulin, while the opposite expression profile is observed in differentiated cells (with the unexplained exception of GBM3 cells that retained Nestin expression also after differentiation). #$p<0.05$, ##$p<0.005$, *$p<0.001$ and **$p<0.0001$ *vs*. the respective CSCs values. The astrocytic differentiation marker GFAP is also overexpressed in differentiated cells from all three GBMs compared to the respective CSC-enriched cultures (see Figure 2 A for GBM1 and Suppl. Figure 4 for GBM 2 and 3).

D) Tumorigenicity of CSCs from GBM1 grown as a monolayer on matrigel after orthotopic implantation in NOD-SCID mice. Pictures show the extent of the tumours 180 days after xenografting different numbers of cells. Tumour area is indicated by the dotted line. Yellow arrowheads highlight the invasion of the contra-lateral hemisphere.

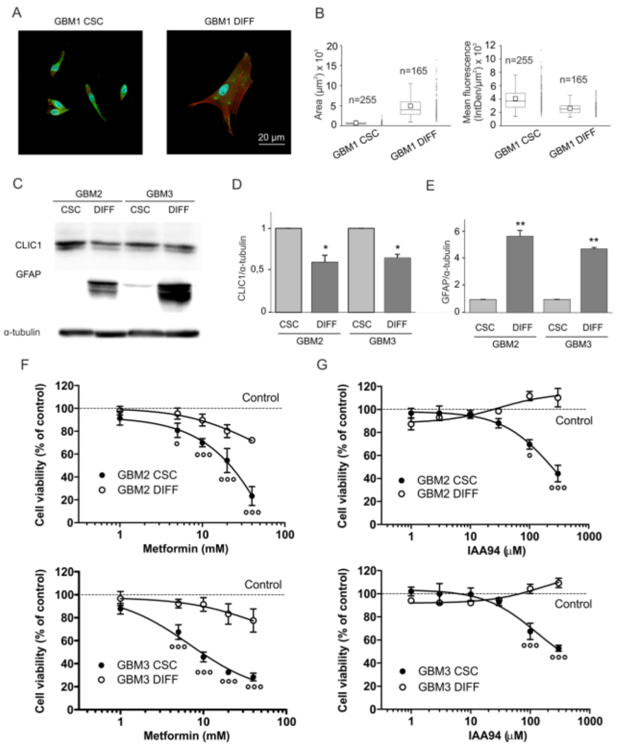

Supplementary Figure 4: **Effects of GBM CSC differentiation on CLIC1 expression.**

A) Representative morphological modifications induced in GBM1 CSCs upon growth factor deprivation and FCS-dependent differentiation. Micrographs show staining with phalloidin (red) to highlight the cell shape and indirect immunofluorescence with anti-CLIC1 antibody (green). A marked reduction of CLIC1 staining is observed.

B) Upon differentiation, GBM1 cells show a marked increase in their surface area compared to undifferentiated CSCs, while the mean fluorescence intensity of CLCI1 staining is significantly reduced, as shown by the plots summarising the immunocytochemistry data.

C) GBM2 and GBM3 cell differentiation causes down-regulation of CLIC1 expression and up-regulation of GFAP, evaluated by Western blot experiments. Similar results were also observed in GBM1 cells (see Figure 2).

D and E) Quantification by densitometric analysis of replica experiments (n=3) for the data reported in (C). Band intensities were normalized to the corresponding a-tubulin signal and are expressed as arbitrary units (A.U.) referred to CSC protein levels (assumed as value 1). *$p< 0.05$ and **$p< 0.001$ vs. respective CSCs (t test).

F) Dose-response curves (1-40 mM) of the effects of metformin on GBM2 and 3 CSC (filled circles) and differentiated (empty circles) cell viability, measured by MTT assays. The assays were run in quadruplicate for each point, data are expressed as mean of n=3 independent experiments; percentage of inhibition was calculated *vs.* untreated controls. A statistically significant reduction in cell viability in CSCs was observed (GBM2: $p<0.001$ for all metformin concentrations *vs*. control; GBM3: $p<0.001$ for 1 mM metformin, $p<0.0001$ for 5-40 mM metformin *vs*. control) (one-way ANOVA, followed Dunnett's post hoc test). In differentiated cells a modest effect was observed when compared to respective untreated controls that was statistically significant only for the highest concentrations tested (20-40mM for GBM2 and 40 mM for GBM3, $p<0.01$). Conversely, at all concentrations tested, the effects of metformin on both GBM2 and GBM3 CSCs were statistically different from those observed in differentiated cells (°$p<0.05$, °°$p<0.01$, °°°$p<0.001$ *vs.* differentiated cells; one-way ANOVA, Tukey's post hoc test)

G) Dose-response effects of IAA94 (1-300 µM) on cell viability, measured by MTT assays, in GBM2 and 3 CSC (filled circles) and differentiated (empty circles) cells. The assays were run in quadruplicate for each point, and the percentage of inhibition was calculated against vehicle control (n=3). A statistically significant inhibition of cell viability was observed for GBM2 ($p<0.05$ for 30 µM IAA94, $p<0.01$ for 100 µM IAA94, $p<0.001$ for 300 µM IAA94 *vs*. control) and GBM3 ($p<0.01$ for 30 µM IAA94, $p<0.001$ for 100 µM and 300 µM IAA94 *vs*. control, one-way ANOVA, followed by Dunnett's post hoc), while differentiated cells were not affected. Conversely, at 100 and 300 µM IAA94, the effects on CSCs were statistically different from those observed in differentiated cells (°$p<0.05$, °°°$p<0.001$ vs. differentiated cells; one-way ANOVA, followed by Tukey's post hoc test) Dotted line represents the respective untreated control values.

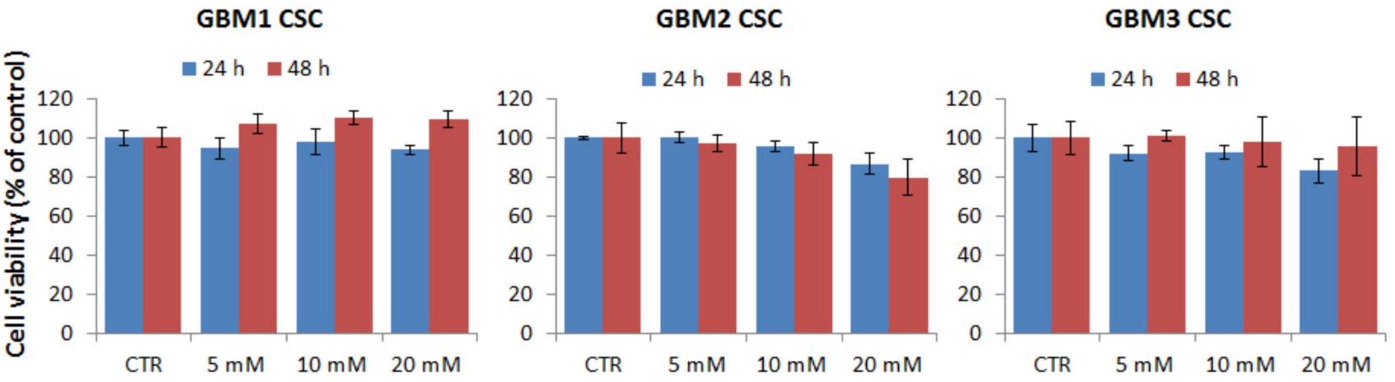

Supplementary Figure 5: **Lack of effects of high concentrations of arginine on GBM 1-3 CSC survival after 24 and 48 hours of treatment.**

The specificity of metformin effects was demonstrated using high concentrations (5-20 mM) of arginine that in MTT experiments was completely ineffective in reducing CSC viability, even after prolonged exposure (48 hours).
